# Herpes simplex 1 encephalitis presenting as a brain haemorrhage with normal cerebrospinal fluid analysis: a case report

**DOI:** 10.1186/1752-1947-2-387

**Published:** 2008-12-17

**Authors:** Effrossyni Gkrania-Klotsas, Andrew ML Lever

**Affiliations:** 1 Addenbrooke's Hospital, University of Cambridge Teaching Hospital NHS Trust, Hills Road, Cambridge CB2 2QQ, UK; 2 Department of Medicine, University of Cambridge, Hills Road, Cambridge CB2 2QQ, UK

## Abstract

**Introduction:**

Herpes simplex encephalitis is a potentially lethal infection that should be recognised as soon as possible. The combination of clinical history and examination, brain computed tomography or magnetic resonance imaging and lumbar puncture has been used to establish a diagnosis.

**Case presentation:**

We present a patient who had a suggestive history but a totally normal lumbar puncture and only evidence of intracerebral haemorrhage in the brain magnetic resonance imaging. Diagnosis was made by using the cerebrospinal fluid polymerase chain reaction for herpes simplex virus.

**Conclusion:**

Herpes simplex encephalitis is being increasingly diagnosed with the availability of new diagnostic techniques. Herpes simplex encephalitis can present with the combination of haemorrhage and normal cerebrospinal fluid. Awareness of this common but, if left untreated, devastating condition should increase.

## Introduction

The rapid diagnosis of central nervous system infection with herpes simplex virus (HSV) is important because of the potential morbidity and mortality associated with the disease as well as the wide availability of acyclovir which has been proven to ameliorate the symptoms. Left untreated, more than 70% of cases of HSV encephalitis (HSVE) are fatal and only approximately 11% of patients recover normal premorbid function [1,2]. Treatment with acyclovir has been proven to reduce mortality to approximately 20% [3,4]. So far, the diagnosis of HSVE has relied on the combination of a compatible clinical scenario, a suggestive brain computed tomography (CT) scan or brain magnetic resonance imaging (MRI) and the examination of the cerebrospinal fluid (CSF) by microscopy, biochemical analysis and polymerase chain reaction (PCR) for the presence of HSV DNA. HSVE has been left undiagnosed in the past, resulting in the patient's demise because of the lack of CSF pleocytosis, a normal CT and the absence of focal features on neurological examination [5].

We present a case that illustrates the importance of the clinical scenario in a patient with atypical findings in imaging studies and a normal CSF examination.

## Case presentation

A 46-year-old man presented to our institution, during the summer months, following referral by another hospital. The patient was in good health until 1 week before admission to our hospital, when he suddenly developed worsening headache, fever and depersonalization. Just before his illness, the patient, who had been residing in the UK for the previous 2 years, had just spent 2 weeks visiting friends and travelling extensively through Georgia, Alabama, Florida and Texas. There were no clues from his contact, sexual or past medical history. He had experienced no bites from animals. He had not spent anytime in the wild and had not driven through desert areas. He was heterosexual and monogamic.

His symptoms worsened during the next 72 hours, when he was admitted to an outside hospital. The patient started experiencing a subjective slowing of mentation, visual hallucinations as well as auditory hallucinations. On direct questioning, he also admitted to subjective perineal paraesthesias and dysaesthesias, without any rash present and the feeling of an erupting "cold sore" on his lip. He also described a vague episode of losing control of his bowel sphincter.

The patient was admitted to the outside hospital and empirical levofloxacin was started for a possible urinary tract infection, based on the presence of fever and perineal dysaesthesias. An HIV test was negative. Conventional blood cultures were negative. A urinary culture grew *Escherichia coli *but the results of the urinary analysis were unavailable. Because of worsening symptoms, on day 6 of the illness, the patient underwent an unenhanced brain CT that was suggestive of a small circumscribed brain haemorrhage. The patient was transferred to our hospital for further management.

On admission, the patient was complaining of subjective fever and headache. He was febrile with a tympanic membrane temperature of 38°C, normotensive and mildly tachycardic. His physical exam was significant for a slow mentation and generalized slowing of his verbal responses. However, he had an appropriate content, although he appeared tearful. Except for generalized mild weakness, his nervous system exam was otherwise normal, with the patient able to subtract serial numbers and perform tandem walking, albeit characteristically slowly. A chest X-ray was normal. Urine analysis and blood cultures were repeated.

**Figure 1:**
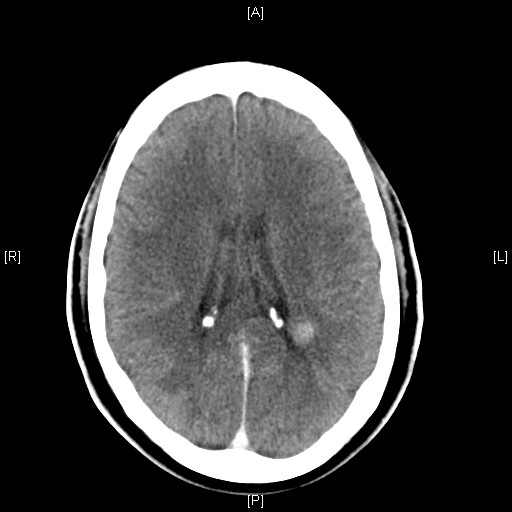
Brain computed tomography scan of the patient on presentation.

The patient was started on ceftriaxone, doxycycline and acyclovir and a CT scan of the patient's brain was obtained after the infusion of iopamidol. The enhanced study revealed a 12 mm focus of increased attenuation consistent with a small haemorrhage adjacent to the posterior body of the left lateral ventricle. No abnormal enhancement was shown. There was no hydrocephalus and the brain parenchyma appeared otherwise normal (Figure 1).

A lumbar puncture performed immediately thereafter revealed an opening CSF pressure of 19 cmH_2_O. The CSF analysis revealed a white blood cell (WBC) count of 0, a red blood cell (RBC) count of 0, a protein level of 0.3 g/L, and a glucose level of 4.3 mmol/L (the synchronous serum value was 5.3 mmol/L). A CSF PCR for viral isolates was ordered, the result of which became available 72 hours after the lumbar puncture had been performed. The CSF PCR was positive for HSV1 DNA and negative for Enterovirus as well as for Varicella Zoster Virus. A viral culture was not performed. Bacterial as well as fungal cultures of the CSF were negative.

**Figure 2:**
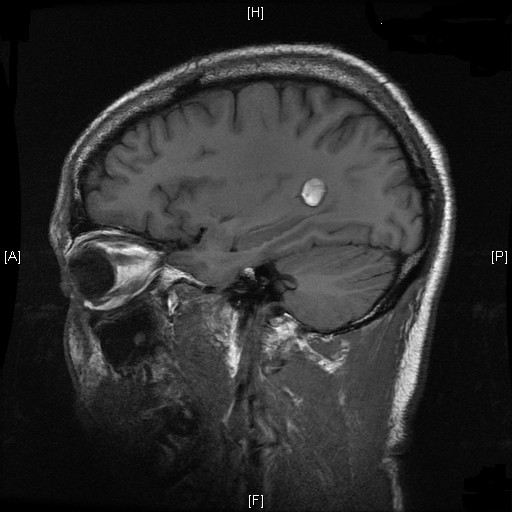
Magnetic resonance imaging of the patient, showing the area of haemorrhage.

The patient's symptoms worsened with increasing confusion before starting to improve on day 3 of the acyclovir (day 9 of the illness). A repeat HIV test was negative, both for antibodies for HIV 1/2 by ELISA and for antigens/antibodies by enzyme immunoassay. An MRI of the brain performed after injection of gadolinium on day 2 of acyclovir (day 8 of the illness) again revealed the small haemorrhage, unchanged in dimensions. No other lesions were identified and the remainder of the parenchyma appeared normal (Figures 1 and 2). The patient remained afebrile for the rest of his stay in hospital while his neurological symptomatology improved. An EEG was not performed. The patient was discharged to the outside hospital in very good health on day 7 of acyclovir, having returned to his premorbid mental condition (day 13 of the illness).

## Discussion

The presence of a totally normal CSF analysis in HSVE is unusual, especially in non-immunocompromised patients. In a study presenting only atypical forms of HSVE [5], only one patient out of 24 had a normal CSF examination, performed during day 1 of the illness. The presence of RBC in the same CSF specimen is not mentioned. A subsequent CSF examination of the same patient performed on day 10 of the illness revealed a mononuclear pleocytosis with accompanying high protein. It is noteworthy that the patient had a normal CT scan of the brain without contrast on day 1 and a subsequent MRI on day 4 that demonstrated an increased signal involving both grey and white matter within a diffusely swollen temporal lobe. In a study of PCR-positive HSVE [6], no patients had < 5 WBC/mm^3^, 4 patients (25%) had 5–50 WBC/mm^3^, 11 patients (68.7%) had 51–500 WBC/mm^3 ^and one patient (6.3%) had > 500 WBC/mm^3^. In a more recent study [7] comparing HSVE with HSVM cases, the HSVE cases had a mean CSF leukocyte count of 202 (range of 2–667 WBC/mm^3^), a mean RBC count of 2518 (0–27,556 RBC/mm^3^) and a mean protein level of 73 (22–146 mg/dl). Six patients in the HSVE group had ≤3 RBC/mm^3^.

The presence of a haemorrhagic lesion in HSVE as the only abnormal finding during radiographic imaging is also very rare. The lack of background abnormalities is rare as well: in a case series of patients with HSVM or HSVE documented by CSF PCR for HSV DNA [7], 14/15 patients with HSVE had a brain CT or MRI positive for frontal lobe and/or temporal lobe involvement and 1/15 patients had only evidence of thalamic involvement. The exact nature of involvement was not described in the study. In an earlier study published in 1997 [6], 33.3% of patients with PCR-positive HSVE had a normal CT of the brain and two-thirds of the rest had a temporal abnormality. CT scan abnormalities included low-density lesions, oedema, contrast enhancement and, less frequently, haemorrhage. The exact percentage of haemorrhagic lesions is not stated in the study. One patient (11.1%) had a normal MRI of the brain and all of the rest of the patients (8 patients, 88.9%) had temporal abnormalities more commonly hyperintensity lesions in the T2-weighted images in one or both inferomedial regions of the temporal lobes, which usually extended to the insular cortex. Lesions of the gyrus rectus (3 patients), cingulated gyrus (2 patients) and basal ganglia (1 patient) were less frequently seen. The possibility that the haemorrhage identified with MRI was an incidental finding and unrelated to HSVE was entertained: on balance, though, because the patient had no history of hypertension, headaches or a personal/family history of arteriovenous malformations, we believe that the haemorrhage was a direct result of the HSVE. The radiographic appearances were also not suggestive of a cavernoma or an arteriovenous malformation.

The validity of the positive HSV PCR in the CSF has been questioned in the past because the HSV genome is found in the trigeminal ganglion in 85% to 90% of unselected autopsies [8]. Although it is theoretically possible that a positive HSV PCR in the CSF could represent asymptomatic latency in the nervous tissue, in the absence of reactivation, it would be difficult to explain how the virus reaches the CSF from the ganglia or brain. Also, studies have shown that the HSV PCR in the CSF has a high specificity, making false positive results quite unlikely. In this patient, where a compatible clinical scenario was present, we are confident that this represents a true result.

An electroencephalogram would have been useful in the diagnosis and management of HSVE in this patient. Unfortunately, one was not performed.

## Conclusion

In the past, mild or atypical forms of HSVE were not included in studies because an autopsy or a brain biopsy were necessary. Enrolment in clinical trials of treatment for HSVE sponsored by the Collaborative Antiviral Study Group of the National Institute of Allergy and Infectious Diseases (CASG-NIAID) required patients to have an acute febrile encephalopathy with disordered mentation, focal cerebral signs, evidence of localization by diagnostic procedures and CSF findings compatible with a viral infection [2,4]. Since the widespread availability of HSV DNA PCR, more atypical or milder cases are now being identified. Many clinical laboratories also pre-screen their CSF samples for subsequent examination by PCR based on studies [9] that have shown that laboratory costs and workload can be substantially when only CSFs with abnormalities are tested. This case proves that very atypical cases might be missed early in their presentation if the laboratory is not alerted to the possibility of HSVE. A HSVE presentation with acellular CSF remains rare and a presentation with haemorrhage as the only radiographic evidence of the disease is equally unusual. Clinical suspicion should remain the most important criterion for early initiation and appropriately timed discontinuation of antiviral treatment. Neither the CSF cell counts nor the brain MRI should be relied upon as sole criteria to exclude a diagnosis of HSVE in the presence of a very suggestive clinical scenario until the CSF PCR is available.

## Abbreviations

ALT: alanine-aminotransferase; CSF: cerebrospinal fluid; CT: computed tomography; EEG: electroencephalogram; ELISA: enzyme linked immunosorbent assay; HIV: human immunodeficiency virus; HSV: Herpes Simplex Virus; HSVE: Herpes Simplex Virus Encephalitis; HSVM: Herpes Simplex Virus Meningitis; MRI: magnetic resonance imaging; PCR: polymerase chain reaction; RBC: red blood cells; WBC: white blood cells

## Consent

Written informed consent was obtained from the patient for publication of this case report and any accompanying images. A copy of the written consent is available for review by the Editor-in-Chief of this journal.

## Competing interests

The authors declare that they have no competing interests.

## Authors' contributions

EGK patient care, data analysis, literature review, drafting manuscript. AMLL patient care, revising manuscript
